# Early Phases of COVID-19 Are Characterized by a Reduction in Lymphocyte Populations and the Presence of Atypical Monocytes 

**DOI:** 10.3389/fimmu.2020.560330

**Published:** 2020-12-09

**Authors:** Andrea Lombardi, Elena Trombetta, Alessandra Cattaneo, Valeria Castelli, Emanuele Palomba, Mario Tirone, Davide Mangioni, Giuseppe Lamorte, Maria Manunta, Daniele Prati, Ferruccio Ceriotti, Roberta Gualtierotti, Giorgio Costantino, Stefano Aliberti, Vittorio Scaravilli, Giacomo Grasselli, Andrea Gori, Laura Porretti, Alessandra Bandera

**Affiliations:** ^1^ Infectious Diseases Unit, Foundation IRCCS Ca’ Granda Ospedale Maggiore Policlinico, Milano, Italy; ^2^ Flow Cytometry and Cell Sorting Laboratory, Clinical Laboratory, Foundation IRCCS Ca' Granda Ospedale Maggiore Policlinico, Milano, Italy; ^3^ Centre for Multidisciplinary Research in Health Science (MACH), University of Milano, Milano, Italy; ^4^ Translational Medicine and Biobank Unit, Department of Transfusion Medicine and Hematology, Foundation IRCCS Ca' Granda Ospedale Maggiore Policlinico, Milano, Italy; ^5^ Department of Transfusion Medicine and Hematology, Foundation IRCCS Ca' Granda Ospedale Maggiore Policlinico, Milano, Italy; ^6^ Clinical Laboratory, Foundation IRCCS Ca' Granda Ospedale Maggiore Policlinico, Milano, Italy; ^7^ Department of Pathophysiology and Transplantation, University of Milano, Milano, Italy; ^8^ Angelo Bianchi Bonomi Hemophilia and Thrombosis Center, Foundation IRCCS Ca' Granda Ospedale Maggiore Policlinico, Milano, Italy; ^9^ Emergency Unit, Foundation IRCCS Ca' Granda Ospedale Maggiore Policlinico, Milano, Italy; ^10^ Respiratory Unit and Cystic Fibrosis Adult Center, Internal Medicine Department, Foundation IRCCS Ca' Granda Ospedale Maggiore Policlinico, Milano, Italy; ^11^ Departement of Anaesthesia, Critical care and Emergency, Foundation IRCCS Ca' Granda Ospedale Maggiore Policlinico, Milano, Italy

**Keywords:** COVID-19, SARS-CoV-2, peripheral blood mononuclear cells, immune profiling, inflammation, monocytes

## Abstract

**Background:**

Severe acute respiratory syndrome coronavirus 2 is a recently discovered pathogen responsible of coronavirus disease 2019 (COVID-19). The immunological changes associated with this infection are largely unknown.

**Methods:**

We evaluated the peripheral blood mononuclear cells profile of 63 patients with COVID-19 at diagnosis. We also assessed the presence of association with inflammatory biomarkers and the 28-day mortality.

**Results:**

Lymphocytopenia was present in 51 of 63 (80.9%) patients, with a median value of 720 lymphocytes/µl (IQR 520-1,135). This reduction was mirrored also on CD8+ (128 cells/µl, IQR 55-215), natural killer (67 cells/µl, IQR 35–158) and natural killer T (31 cells/µl, IQR 11–78) cells. Monocytes were preserved in total number but displayed among them a subpopulation with a higher forward and side scatter properties, composed mainly of cells with a reduced expression of both CD14 and HLA-DR. Patients who died in the 28 days from admission (N=10, 15.9%), when compared to those who did not, displayed lower mean values of CD3+ (337.4 cells/µl vs 585.9 cells/µl; p=0.028) and CD4+ cells (232.2 cells/µl vs 381.1 cells/µl; p=0.042) and an higher percentage of CD8+/CD38+/HLA-DR+ lymphocytes (13.5% vs 7.6%; p=0.026).

**Discussion:**

The early phases of COVID-19 are characterized by lymphocytopenia, predominance of Th2-like lymphocytes and monocytes with altered immune profile, which include atypical mononuclear cells.

## Introduction

Severe acute respiratory syndrome coronavirus 2 (SARS-CoV-2) is a new beta coronavirus identified in China in December 2019 which is responsible of coronavirus disease 2019 (COVID-19) (1). The virus rapidly spread worldwide, and it is responsible of a pandemic which is exerting a tremendous pressure on national health systems (2).

Given the novelty of this virus, how the immune system deals with it is largely unknown. Preliminary studies from China highlighted a reduction of lymphocytes count, particularly of CD4+ T and CD8+ T cells, an increase of the neutrophils-to-lymphocytes ratio (NLR), a concomitant decrease in interferon gamma (IFN-γ) production and an association with elevated inflammatory markers (3–5). An excessive pro-inflammatory cytokines release profile was confirmed also by a transcriptomic study performed on peripheral blood mononuclear cells (PBMC) (6). Also innate immunity cells are involved. Indeed, Zheng and colleagues demonstrated an upregulation of the inhibitory receptor NKG2A expression on natural killer cells (NK) and cytotoxic lymphocytes (CTLs) in COVID-19 patients with a compromised degranulation capacity and a reduced production of IFN-γ, IL-2, granzyme B and tumor necrosis factor α (TNF-α) (7). Finally, preliminary unpublished reports described the presence of peripheral blood monocytes with morphologic and phenotypic changes displaying macrophage markers. The severity of the alterations occurring to these atypical monocytes was correlated with patient outcome (8).

A better knowledge of the immune response against the virus is mandatory, since it has been postulated that severe forms of the disease are associated with a cytokine storm, where monocytes/macrophages play a central role (9–12). As a consequence, several trials evaluating the efficacy of immunosuppressive or immunomodulatory drugs (tocilizumab, anakinra, sarilumab, eculizumab, ruxolitinib, fingolimod, emapalumab, tofacitinib, meplazumab) are currently undergoing worldwide (13).

In attempt to provide our contribution to the field, we designed the present study describing PBMC’s characteristics and basic inflammatory markers values in a cohort of Italian patients who were diagnosed of SARS-CoV-2 infection and were hospitalized.

## Materials and Methods

### Study Population

We enrolled patients consecutively admitted to the Foundation IRCCS Ca’ Granda Ospedale Maggiore Policlinico, a University Hospital in Milano, Italy, diagnosed with COVID-19 in the period from March 17 to April 3, 2020. Diagnosis of COVID-19 was defined by the presence of compatible signs/symptoms (fever, cough, coryza, myalgia, diarrhoea, dyspnoea, tachypnoea, ageusia, anosmia) and a positive nasopharyngeal swab for SARS-CoV-2 detected through real-time reverse transcription-polymerase chain reaction (rRT-PCR). Serum levels of inflammatory biomarkers were assessed as standard clinical practice. Flow cytometry analysis was performed on fresh blood left-over of samples collected at hospital admission for clinical purposes. We analyzed only blood samples collected in the first 48 h since diagnosis. Samples of fresh blood left-over from local blood donor bank were also employed as healthy controls. The study was approved by the Institutional Review Board Milano Area 2 (#358_2020) and was conducted in accordance with the Helsinki Declaration.

### Inflammatory Biomarkers Assessment and Clinical Data Collection

Procalcitonin (PCT), ferritin and interleukin-6 (IL-6) were measured with electro-chemiluminescent immunoassays (ECLIA) on a Roche Cobas e801 instrument (Roche Diagnostics, Monza, Italy). C-reactive protein (CRP) was measured with an immunoturbidimetric method and lactate dehydrogenase (LDH) with the International Federation of Clinical Chemistry (IFCC) optimized method on a Roche Cobas c702 instrument (Roche Diagnostics, Monza, Italy).

For each patient, the P/F ratio [partial pressure of oxygen (PaO_2_)/fraction of inspired oxygen (FiO_2_)], was calculated at the admission to the hospital. The 28-day mortality was collected from electronic medical records.

### SARS-CoV-2 Detection

Two different methods were used for viral detection. The first one consisted in Seegene Inc reagents (Seoul, Korea), RNA extraction with STARMag Universal Cartridge kit on Nimbus instrument (Hamilton, Agrate Brianza, Italy) and amplification with Allplex® 2019-nCoV assay, while the second employed a GeneFinder® COVID-19 Plus RealAmp Kit (OSANG Healthcare, Anyangcheondong-ro, Dongan-gu, Anyang-si, Gyeonggi-do, Korea) on ELITech InGenius® instrument (Torino, Italy). Both assays identify the virus by multiplex rRT-PCR targeting three viral genes (E, RdRP and N).

### Flow Cytometry Analysis

Fresh peripheral blood samples were processed within 24 h from collection for the following evaluations: i) classical lymphocyte subpopulations count, ii) CD4+ T-cells polarization (14–16), iii) lymphocytes activation status, iv) monocyte subpopulations, and v) NK-cell subsets. For this purpose, we employed the monoclonal antibodies (BD Biosciences, San Jose, CA) reported in **Supplementary Table 1**. **Supplementary Figures 1 to 5** show the surface markers and gating strategy employed to identify different immune cells. Each antibody was titrated individually and the optimal dilution for a given staining of 100 µl volume of whole blood was determined by comparison with the isotype matched controls. After the cell staining, erythrocytes were lysed with lysis buffer (Pharm Lyse, BD Biosciences). Following the washing, samples were acquired using a BD FACSLyric flow cytometer equipped with three lasers: a 405 nm violet laser, a 488 nm blue laser, and a 647 nm red laser. For each tube we set a stopping gate criterion of 50,000 events in the lymphocyte gate, forward scatter (FSC) versus side scatter (SSC), and the data were analysed using FACSSuite and FlowJo softwares (BD Biosciences). An automatic standard compensation was applied for each acquisition. Internal quality assurance procedures included BD cytometer setup and tracking beads, according to the manufacturer’s instructions.

### Statistical Analysis

Descriptive statistics were performed for all the variables assessed in the study population. The Spearman test and linear regression were used to examine correlations. The Friedmann test followed by Dunn’s correction for multiple comparisons and multiple t test followed by Holm-Sidak correction for multiple comparisons were employed to assess differences among groups. Parametric tests were employed for continuous variables, whereas non-parametric test for those not-normally distributed (Kolmogorov-Smirnov test). A *p* value <0.05 was deemed statistically significant. All the analysis was performed with GraphPad Prism 8 (GraphPad Inc, USA).

## Results

### Demographic and Clinical Characteristics and Biochemical Values

Sixty-three patients with confirmed SARS-CoV-2 infection were enrolled, on average 5.3 days (SD 2.9) after symptoms onset. The median P/F ratio at admission was 170 mmHg. Hypoxemia was severe (P/F < 100 mmHg) in 13 patients (20.6%), moderate (P/F 100–200 mmHg) in 13 (20.6%) and mild (P/F 200–300 mmHg) in 23 (36.5%), while the remaining 14 patients had a P/F > 300 mmHg. Overall, the 28-day mortality rate was 15.9% (10/63). Demographic and clinical characteristics and inflammatory biomarker values are shown in **Table 1**. Overall, all the inflammatory markers considered were above the reference intervals.

**Table 1:** Demographic and clinical characteristics and inflammatory biomarker levels of 63 patients with COVID-19 at diagnosis.

Variable	Value	Reference values	
**Men, n (%)**	48 (76.2)		
**Age, mean (SD)**	59.1 (13.7)		
**Days since symptoms appearance, mean (SD)**	5.3 (2.9)		
**P/F ratio (mmHg)**	170 (107–290)	>300	*
**CRP (mg/dl)**	11.95 (7.43–19.02)	<0.5	*
**Ferritin (µg/L)**	1,503 (724–2,887)	30–400	*
**LDH (U/L)**	335 (273–424)	135–225	*
**IL-6 (ng/L)**	81.95 (28.9–112.8)	0–10	*
**PCT (µg/L)**	0.33 (0.16–1.46)	0.02–0.06	*
**Fibrinogen (mg/dl)**	571 (470–722)	165–300	*
**D-dimer (µg/L)**	1,479 (678–2,529)	<500	*
**ALT (U/L)**	41 (28–69)	9–59	

All data are reported as median with interquartile range (IQR) except when stated otherwise. *Values outside of normality range. [P/F ratio, partial pressure of oxygen (PaO_2_)/fraction of inspired oxygen (FiO_2_); CRP, C-reactive protein; LDH, lactate dehydrogenase; IL-6, interleukin 6; PCT, procalcitonin; ALT, alanine aminotransferase].

### White Blood Cells Total Count and Subpopulations

At diagnosis of SARS-CoV-2 infection, median values of white blood cell (WBC), monocyte and neutrophil were within the reference intervals employed in our Centre. Instead, median lymphocyte count was below the lower reference limit. Overall, lymphocytopenia was found in 51 of the 63 patients (80.9%). Consequently, the NLR was oriented toward high values, well above the reference intervals of 0.78–3.53 (**Table 2**) (17).

**Table 2:** White blood cell count, leukocyte subpopulations and reference values employed in our center.

Variable	Value	Reference values	
**White blood cells (cells/µl)**	6,520 (5,015–9,200)	4,800–10,800	
**Monocytes (cells/µl)**	330 (195–510)	300–600	
**Lymphocytes (cells/µl)**	720 (520–1,135)	1,200–3,400	*
**Neutrophils (cells/µl)**	5,070 (3,785–8,105)	1,500–6,500	
**Neutrophils/Lymphocytes ratio**	7.5 (4.39–14.02)	0.78–3.53	*
**CD3+ lymphocytes (cells/µl)**	500 (273–728)	700–2,100	*
**CD4+ lymphocytes (cells/µl)**	342 (192–451)	300–1,400	
**CD8+ lymphocytes (cells/µl)**	128 (55–215)	200–900	*
**CD4+/CD8+ ratio**	2.93 (1.9–3.7)	1–3.6	
**CD19+ lymphocytes (cells/µl)**	120 (54–182)	100–500	
**Natural killer cells (cells/µl)**	67 (35–182)	90–600	*
**Natural killer T cells (cells/µl)**	31 (11–73)	143 (median value)	*
**Platelets (10^3^ cells/µl)**	247 (162–318)	130–400	

All data are reported as median with interquartile range (IQR). * Values outside reference intervals.

### Lymphocyte Subpopulations

**Figure 1:**
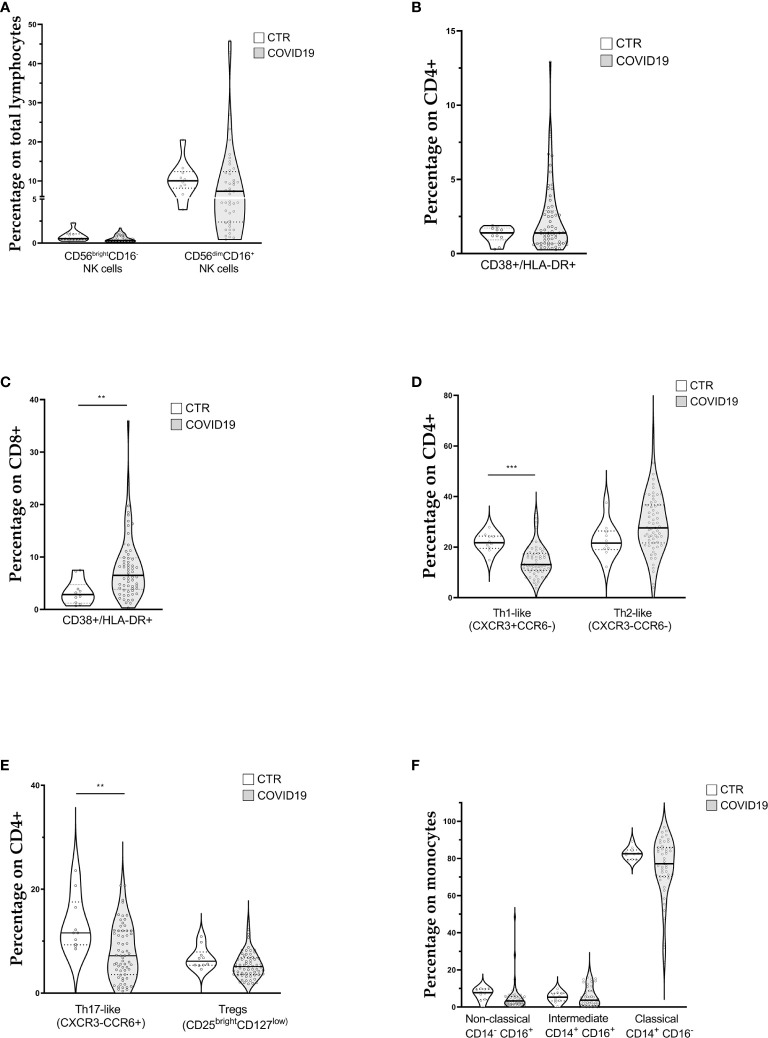
Violin plots showing lymphocyte and monocyte subpopulations (reported as median value) assessed in 63 patients with COVID-19 at diagnosis and 10 controls (CTR). **(A)** Natural killer cell subpopulations **(B)** CD4+ cells activation; **(C)** CD8+ cells activation **(D, E)** CD4+ cells polarization; **(F)** Monocytes subpopulations. [Th1-like (CXCR3+CCR6-): Type 1 T helper-like cells; Th2-like (CXCR3-CCR6-): Type 2 T helper-like cells; Th17-like (CXCR3-CCR6+): T helper 17-like cells; Tregs: regulatory T cells; NK: natural killer cells]. t-test was used to calculate statistical difference. (** p value <0.01; *** p value <0.005).

Lymphocyte subpopulations mirrored the general feature of lymphocytopenia. Median values of CD3+ and CD8+ lymphocyte counts were below the lower reference limit (**Table 2**). Likewise, the two groups of cytotoxic lymphocytes (CTLs) belonging to the innate immunity, NK (CD3-CD16+CD56+) and NKT (CD3+CD16+CD56+) cells, were below the lower reference limits (**Table 2**). Regarding the frequencies of NK subsets, we did not detect any statistical difference of CD56^bright^CD16- (immature) and CD56^dim^CD16+ (mature) NK cells among total lymphocytes between patients and healthy donors (CTR) (**Figure 1A**).

Concerning T lymphocytes activation, assessed through the expression of CD38 and HLA-DR on CD4+ (**Figure 1B**) and CD8+ cells (**Figure 1C**), we found a significant increase of activated CTLs (6.8% IQR 3.9–10.1 vs 2.9% IQR 1.5–5.7) in COVID patients.

**Figures 1D, E** show CD4+ cell polarization The relative median frequencies of Th1-like (CXCR3+CCR6-), Th2-like (CXCR3-CCR6-), Th17-like (CXCR3-CCR6+), and Treg (CD25++CD127low) lymphocytes were 13.1% (IQR 10.8–17.6), 27.6% (IQR 21.7–36.7), 7.2% (IQR 3.6–12), and 5.1% (IQR 3.6–6.8), respectively. Reference values for the same cell populations were 21.8% (IQR 20–24), 21.6% (IQR 19–25), 11.6% (IQR 9–15), 6.2% (IQR 5–7) of CD4+ cells for Th1-like, Th2-like, Th17-like, and Treg lymphocytes, respectively. COVID-19 patients showed a statistically significant reduction in percentage of Th1-like and Th17-like cells. The Th1/Th2 ratio (0.47, IQR 0.36–0.65 vs. 1.0, IQR 0.9–1.1) was altered, whereas the Treg/Th17 ratio was not influenced (0.62, IQR 0.5–1.2 vs 0.8, IQR 0.5–0.9).

### Monocytes

The median frequencies of monocyte subpopulations are displayed in **Figure 1F**. Overall, relative subpopulations were 3.3% (IQR 1.66–5.7), 3.7% (IQR 0.8–8.7), and 77.2% (IQR 70.28–85.9) for non-classical (CD14^dim^CD16+), intermediate (CD14+CD16+), and classical monocytes (CD14+CD16-), respectively. Reference intervals for the above monocyte subpopulations were 7.7% (IQR 5–10), 5.3% (IQR 4–7), and 82.6% (IQR 80–84) for non-classical, intermediate and classical monocytes, respectively. Finally, the median percentage of HLA-DR+ monocytes was 83.4% (IQR 75.2–92.3), below our internal control reference value of 96% (IQR 94–98).

**Figure 2:**
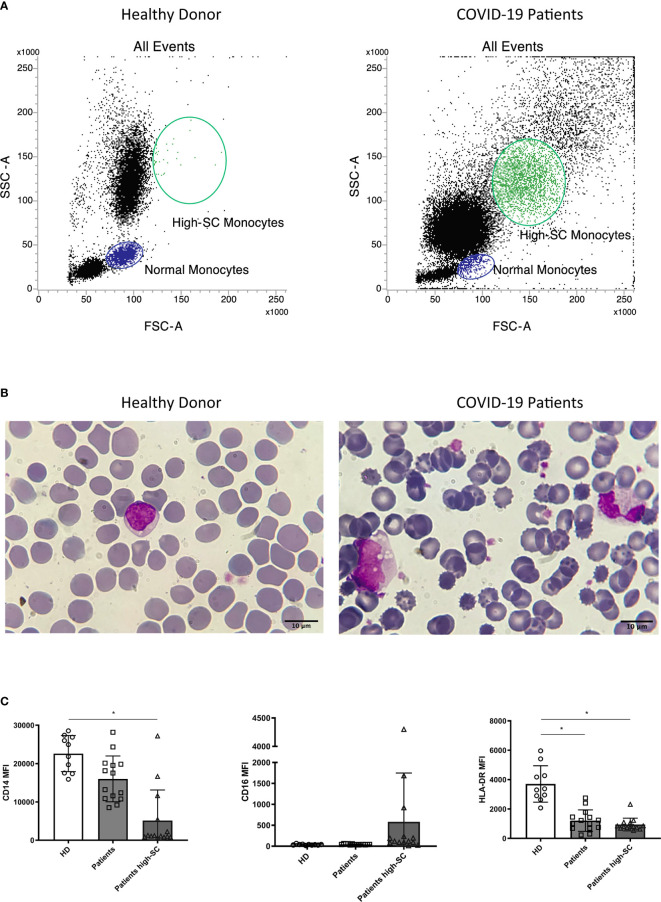
Morphologic and phenotypic differences between monocytes from a healthy donor (left) and a COVID-19 patient (right): **(A)** flow cytometry dot plot showing normal and high-SC monocytes; **(B)** monocytes from May-Grunwald Giemsa stained blood smears (100X magnification, Leica Microscope DMLS); **(C)** histograms showing median fluorescence intensity (MFI) of CD14, CD16, and HLA-DR on monocytes from healthy donors (HD, n=10), normal and atypical monocytes from COVID-19 patients (n=14). Non parametric Kruskal Wallis test was used to determine significant difference. Kolmogorov-Smirnov test was used to assess normal distribution of samples. (* *p* value <0.005).

Morphological identification of monocytes in COVID-19 patients became challenging due to altered scatter properties. Furthermore, we noticed distinct CD4^dim^ cell population with high FSC and SSC (**Figure 2A**). Blood smear examination confirmed the presence of atypical monocytes with vacuoles in their cytoplasm (**Figure 2B**). Therefore, we modified flow cytometer parameters with reduced photomultiplier tube values in order to analyze better these peculiar monocytes (high-SC). We performed this analysis in a subgroup of 14 patients and we considered significant a value of high-SC > 2% of total monocytes. Overall, the high-SC population was present in 11/14 (79%) patients where they represented a variable fraction of total monocytes (range 2%–14%).

Moreover, to better understand the differences in the phenotypic signature between normal and high-SC monocytes in COVID-19 patients and in healthy donors (n=10), we compared the Median Fluorescence Intensity (MFI), a parameter proportional to antigen density, of CD14, CD16, and HLA-DR in these monocyte populations. Overall, the MFI of CD14 and HLA-DR on high-SC monocytes was lower than those of healthy donors (*p*<0.05). Regarding HLA-DR, we found a significant reduction on COVID-19 patients’ monocytes with normal scatter properties (*p*<0.05). For a better characterization of these atypical cells, we compared their CD14 and HLA-DR expression with normal monocytes and granulocytes of the same patient. As shown in **Supplementary Figure 7**, high-SC monocytes expressed similar marker profile of non-classical and classical monocytes.

Finally, we observed a rising trend of CD16 MFI, although not significant, in high-SC monocytes compared to normal monocytes from COVID-19 patients and healthy donors (**Figure 2C**).

### Correlations With Inflammatory Biomarkers

We also assessed the relationship between the different variables evaluated in our cell populations and inflammatory biomarkers. Interestingly, the IL-6 values of and the monocyte counts were not correlated (R=0.01; *p*=0.60). (**Supplementary Figure 6**). Also, all other variables did not show any significant correlation.

### Variables Associated With Death

Finally, we stratified all the results according to 28-day mortality. Patients who died within 28 days from admission had higher age, LDH values, CD4/CD8 ratio and percentage of CD38+/HLA-DR+ CTLs compared to those alive (**Table 3**). Instead, they had lower P/F ratio, CD3+ and CD4+ lymphocyte counts. All other variables did not show any significant difference.

**Table 3:** Variables with significant differences between those who died in the 28 days from admission and those who did not.

	*p* value	Mean dead	Mean alive	Difference	SE of difference	t ratio
**Age (years)**	0.002	71.3 (n=10)	56.8 (n=53)	14.5	4.4	3.3
**LDH (U/L)**	0.024	484.2 (n=10)	338.2 (n=20)	145.9	61.2	2.4
**P/F admission**	0.043	138.1 (n=10)	204.6 (n=53)	-66.5	32.2	2.1
**CD3+ (cells/µl)**	0.028	337.4 (n=10)	585.9 (n=49)	-248.6	110.5	2.2
**CD4+ (cells/µl)**	0.042	232.3 (n=10)	381.1 (n=49)	-148.8	71.6	2.1
**CD4/CD8 ratio**	0.015	5.4 (n=10)	3.0 (n=49)	2.4	0.9	2.5
**CD38+/HLA-DR+ on CD8+ cells (%)**	0.026	13.5 (n=10)	7.6 (n=52)	5.8	2.5	2.3

[LDH, lactate dehydrogenase; P/F, partial pressure of oxygen [PaO_2_]/fraction of inspired oxygen (FiO_2_)] Multiple t-test with Holm-Sidak correction for multiple comparisons.

## Discussion

In our cohort of patients hospitalized for COVID-19, we observed a reduction of circulating lymphocytes, both in terms of total count and specific subpopulations. Precisely, all cytotoxic lymphocytes (CTLs, NK, and NKT cells) were below the lower reference limits. Moreover, activated CD8+ T cells were increased, whereas CD4+ lymphocytes were polarized toward a Th2-like phenotype. Intriguingly, monocytes, although not modified in cell number and distribution, displayed morphological and phenotypical alterations. IL-6 levels were not correlated to monocyte counts. Finally, patients who died within 28 days from admission presented lower counts of CD3+ and CD4+ cells and a higher percentage of activated CTLs.

Our results are in line with those recently reported by several groups regarding a profound reduction of lymphocytes in the context of a preserved total WBC count (4, 10, 18). Of note, in our cohort lymphocytopenia was present in 81% of the enrolled patients, a higher percentage compared to those observed in the works by Chen (72% of severe cases, 10% of moderate cases) and by Huang (63% of all cases) (4, 19). The reduction of cytotoxic lymphocytes, both of innate and adaptive immunity, is a relevant finding. Indeed, in the context of several rheumatologic diseases, cytolytic cells may induce apoptosis in activated macrophages and T cells controlling the inflammatory response (20). An impairment in the cytolytic compartment may result in a overstimulation of the immune system leading to the multiorgan failure and this defect has been linked to elevated levels of IL-6 (21).

Regarding monocytes, Zhang and Zhou reported in their studies (22, 23) a relative increase in intermediate and non-classical monocytes. Instead, we detected normal value of classical and non-classical monocytes and a slightly reduction of intermediate monocytes. It should be underlined that our analysis was performed in samples collected early in the course of the disease, while it is unclear when the samples were collected in the studies conducted in China. Similarly to these studies, a fraction of monocytes observed in our cohort showed not only an increase in cell size but also in cell complexity, probably due to the presence of vacuoles in cytoplasm. We also noted an evident decrease of cell complexity in granulocyte population, that requires further investigation. These high-SC monocytes displayed a decrease of CD14 and HLA-DR antigen density. Notably, we also detected a reduced expression of HLA-DR, either as percentage or fluorescence intensity, on patients’ monocytes with normal scatter properties. The same observation was made by Kuri-Cervantes et al. and by Giamarellos-Bourboulis et al. (10, 24). The latter also demonstrated an inverse correlation between HLA-DR molecules and serum levels of IL-6 (10). This dysregulated monocyte immune profile could be also related to weakened SARS-CoV-2 antigen presentation and defective crosstalk with T lymphocytes (25). It will be interesting to follow this population of atypical monocytes during the course of the disease.

Monocytes, considered the crucial players in the pathogenesis of lung damage caused by the cytokines storm, showed substantial phenotypical alterations already in the early phase of the disease. The mechanism leading to monocytes alteration is unclear, but a direct viral role can be suspected. Indeed, Zhang et al (22) showed how monocytes express ACE2, the entry receptor of SARS-CoV-2, and it has been reported how other viral infections (influenza A virus, vaccinia virus, vesicular stomatitis virus) can trigger rapid and substantial differentiation of monocyte profile toward dendritic cells (25).

Concerning lymphocyte compartment, Zhou and colleagues observed that CD4+ T lymphocytes were rapidly activated to become pathogenic Th1 cells, secreting proinflammatory cytokines. The resulting environment induced inflammatory CD14+CD16+ monocytes with high expression of IL-6 (26). In contrast, in our study the T helper ratio was oriented toward a Th2-like polarization. This could be due to IL-6 effect on the immune response towards Th2, by promoting early IL-4 secretion (27), even though in our cohort we did not detect any correlation between IL-6 values and Th-2 like cells. Likewise, Giamarellos-Bourboulis et al. did not detect an immune response oriented toward a Th1 phenotype in their cohort, with IFNγ values below the detection limit in all the patients (10). We acknowledge that in addition to the surface markers we have considered, employment of CCR4 would have helped better identify the Th2-enriched subset.

Finally, we observed some peculiar results stratifying the variables according to 28-day mortality. Indeed, in addition to the clinical variables which are expected to be linked with the disease outcome (age, P/F ratio, LDH), we observed lymphocytopenia and CTLs activation status more evident in those patients who died. It is hard with the current knowledges to understand the significance of these data. Both lymphocytopenia and lymphocyte activation could be the physiologic consequence of an ongoing viral infection, particularly evident in those with a severe outcome. However, it could be interesting to assess whether lymphocytes of these patients are able to control activated innate immunity and modulate inflammatory response. Indeed, in mice, Kim et al. demonstrated that after a viral infection, an unleashed innate immune response, due to the absence of residential T cells, can also be a direct cause of death (28).

It should be noted that our samples were collected at diagnosis, on average of 5 days from the appearance of symptoms. Considering that the mean incubation time of COVID-19 has been estimated to be 5 days, we can assume that our patients were on average of the 10th day after infection. For this reason, our results could be considered a faithful description of the immunological changes occurring in the early phases of the disease and could complement well the data provided by others. At the same time, we acknowledge that our research has limitations. First, it is a single center study, conducted on a small number of patients. Second, it involved only patients diagnosed in hospital, therefore with a severe disease. Third, we did not investigate the presence and the features of immune cells at alveolar level, where most of COVID-19’s immunopathology occurs.

In conclusion, we present here the first description of immunologic features at diagnosis of a cohort of Italian COVID-19 patients that both confirm already available evidence and add novel elements to the COVID-19 jigsaw. Our data might suggest that, in the early phases of COVID-19, the virus can elicit an inflammatory response leading to a reduction in the number of both cytotoxic lymphocytes (CTL, NK cells, NKT cells) and helper lymphocytes. These changes, which are more pronounced in patients with adverse outcome, could represent the first steps in the cascade leading to the uncontrolled activation of monocytes in the context of cytokines storm. It is possible to speculate that an early initiation of an immunomodulatory treatment might abrogate the immune system alterations leading to monocytes activation-dysfunction and to the consequent lung damage. Nevertheless, further prospective studies are needed to provide a precise picture of the immunologic profile during the course of the disease in order to define better therapeutic approaches and to improve the clinical management of COVID-19 patients.

## “COVID-19 Network” Working Group

**Fondazione IRCCS Ca’ Granda Ospedale Maggiore Policlinico**. Scientific Direction: Silvano Bosari, Luigia Scudeller, Giuliana Fusetti, Laura Rusconi, Silvia Dell’Orto; Department of Transfusion Medicine and Hematology (Biobank): Daniele Prati, Luca Valenti, Silvia Giovannelli; Infectious Diseases Unit: Andrea Gori, Alessandra Bandera, Antonio Muscatello, Davide Mangioni, Laura Alagna, Giorgio Bozzi, Andrea Lombardi, Riccardo Ungaro, Teresa Itri, Valentina Ferroni, Valeria Pastore, Roberta Massafra, Ilaria Rondolini; Angelo Bianchi Bonomi Hemophilia and Thrombosis Center and Fondazione Luigi Villa: Flora Peyvandi, Roberta Gualtierotti, Barbara Ferrari, Raffaella Rossio, Elisabetta Corona, Nicolò Rampi, Costanza Massimo; UOC Internal Medicine, Immunology and Allergology: Nicola Montano, Barbara Vigone, Chiara Bellocchi, Elisa Fiorelli, Valerie Melli, Eleonora Tobaldini; Respiratory Unit and Cystic Fibrosis Adult Center: Francesco Blasi, Stefano Aliberti, Maura Spotti, Edoardo Simonetta, Leonardo Terranova, Francesco Amati, Carmen Miele, Sofia Misuraca, Alice D’Adda, Silvia Della Fiore, Marta Di Pasquale, Marco Mantero Martina Contarini, Margherita Ori, Letizia Morlacchi, Valeria Rossetti, Andrea Gramegna, Maria Pappalettera, Mirta Cavallini, Annalisa Vigni; Cardiology Unit: Marco Vicenzi, Irena Rota. Emergency Unit: Giorgio Costantino, Monica Solbiati, Ludovico Furlan, Marta Mancarella, Giulia Colombo, Giorgio Colombo, Alice Fanin; Acute Internal Medicine: Valter Monzani, Angelo Rovellini, Laura Barbetta, Filippo Billi, Christian Folli; Rare Diseases Center: Marina Baldini, Irena Motta, Natalia Scaramellini; General Medicine and Metabolic Diseases: Anna Ludovica Fracanzani, Rosa Lombardi, Federica Iuculano; Geriatric Unit: Matteo Cesari, Marco Proietti, Laura Calcaterra. **Istituto di Ricerche Farmacologiche Mario Negri IRCCS**: Alessandro Nobili, Mauro Tettamanti, Igor Monti.

## Data Availability Statement

The raw data supporting the conclusions of this article will be made available by the authors, without undue reservation.

## Ethics Statement

The studies involving human participants were reviewed and approved by Comitato Etico Milano Area 2, Fondazione IRCCS Ca’ Granda, Francesco Sforza 28, 20122 Milano. The patients/participants provided their written informed consent to participate in this study.

## Authors Contributions

AL, LP, AB, and AG conceived the study. VC, EP, GL, GC, VS, GG, RG, and MM enrolled the patients. LP, AC, ET, MT, and FC performed the immunologic and biochemical analysis. AL, LP, and AB analyzed the data. AL and LP wrote the first draft. All authors contributed to the article and approved the submitted version.

## Funding

The grant “COVID-19 Biobank” was provided by the Scientific Direction, Foundation IRCCS Ca' Granda Ospedale Maggiore Policlinico, Milano, Italy.

## Conflict of Interest

The authors declare that the research was conducted in the absence of any commercial or financial relationships that could be construed as a potential conflict of interest.
