# Supplementary material for: Early Phases of COVID-19 Are Characterized by a Reduction in Lymphocyte Populations and the Presence of Atypical Monocytes
Source: Front Immunol. 2020 Dec 9;11:560330. doi: 10.3389/fimmu.2020.560330 (PMC7756112; doi:10.3389/fimmu.2020.560330)
Supplement: Supplementary file 1 [file Table_1.docx]

Supplementary Material

**Early phases of COVID-19 are characterized by a reduction of lymphocyte populations and the presence of atypical monocytes**

## Supplementary figures


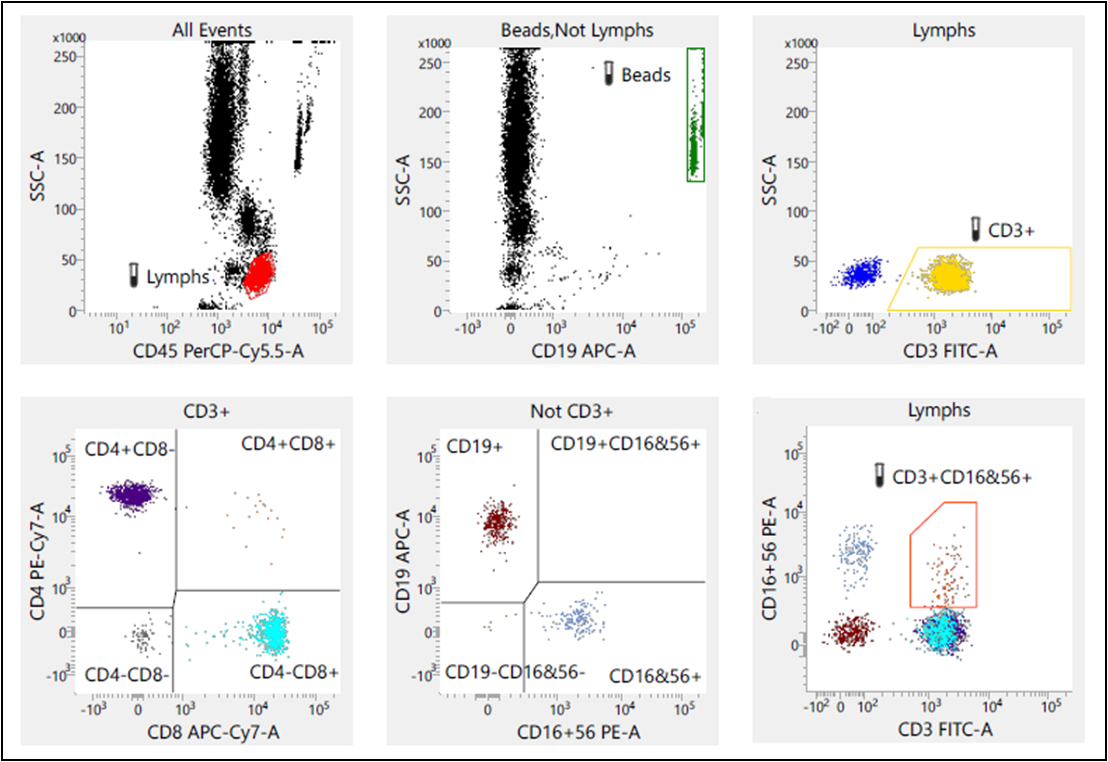


**Supplementary figure 1.** Gating strategy of Multitest 6-color TBNK Kit (BD) for the evaluation of lymphocyte subpopulations. Lymphocytes gate (red) was set on CD45 vs SSC (Side Scatter) dot plot, while bead events for the absolute count (green) were gated, after lymphocytes exclusion, based on their high fluorescence and scatter properties. Lymphocytes were divided in CD3+ cells (T lymphocytes, yellow) and CD3- cells (blue); subsequently T-helper cells (CD4+CD8-, purple) and T cytotoxic cells (CD8+CD4-, light blue) were gated from CD3+ population. From CD3- events, were derived CD19+ cells (B lymphocytes, brown) and CD16+56+ (NK lymphocytes, grey). FACS Suite Clinical software also provide the count of an additional population in the T cell compartment, CD3+CD16+56+.


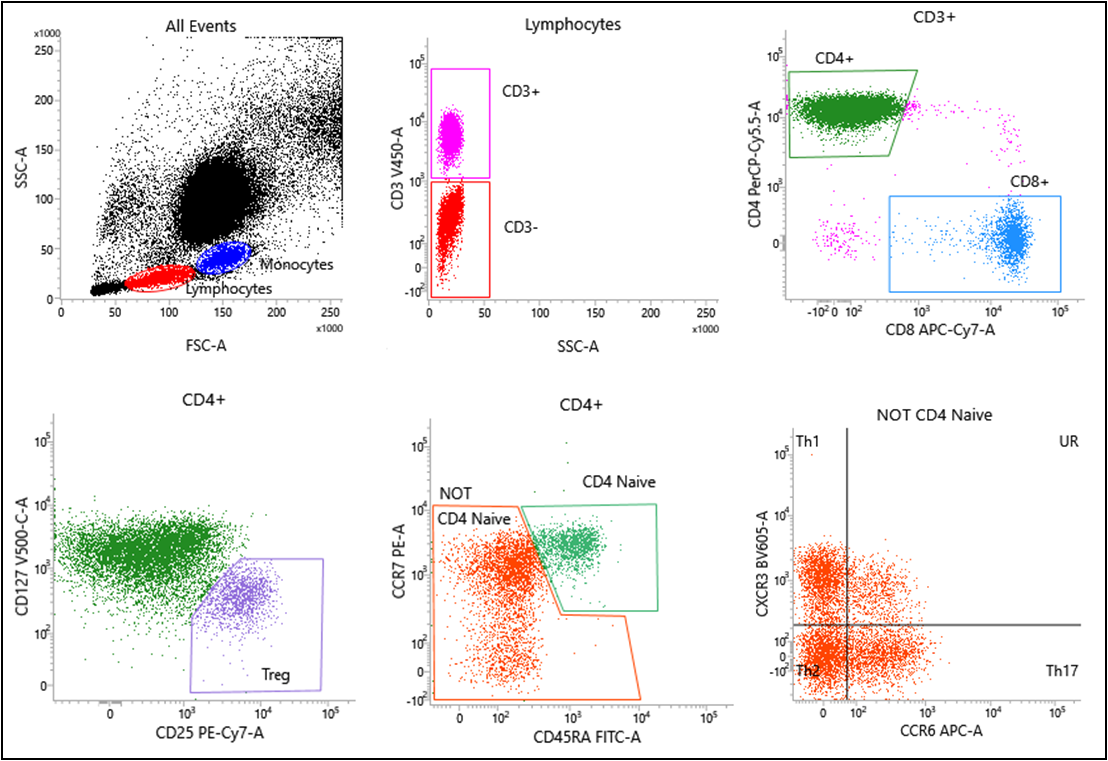


**Supplementary figure 2.** CD4 T cell polarization. Lymphocytes (red) were firstly gated on the basis of their scatter properties (Side Scatter, SSC and Forward Scatter, FSC) and then divided in CD3+ cells (T lymphocytes) and CD3- cells; subsequently T-helper cells (CD4+CD8-) and T cytotoxic cells (CD8+CD4-) were gated in T lymphocyte population. Within the CD4+ cell population were identified T regulatory lymphocytes (Treg, CD25++CD127low, in violet) and, after the exclusion of CD4+ naïve T cell (CD4+CCR7+CD45RA+), we evaluated the distribution of Th1-like (CXCR3+CCR6-), Th2-like (CXCR3-CCR6-) and Th17-like (CXCR3-CCR6+) cell populations.


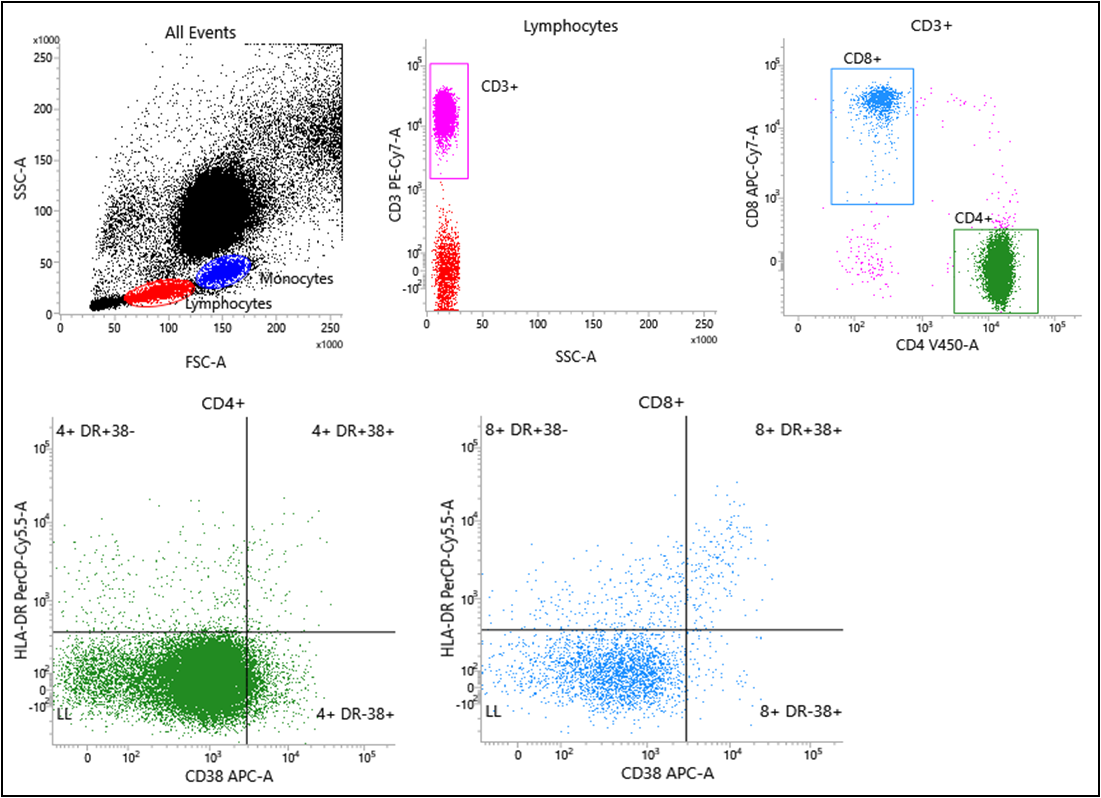


**Supplementary figure 3.** Lymphocyte activation status. For both CD4+ (green) and CD8+ (light blue) lymphocytes was evaluated the expression of the activation markers CD38 and HLA-DR (lower panels).


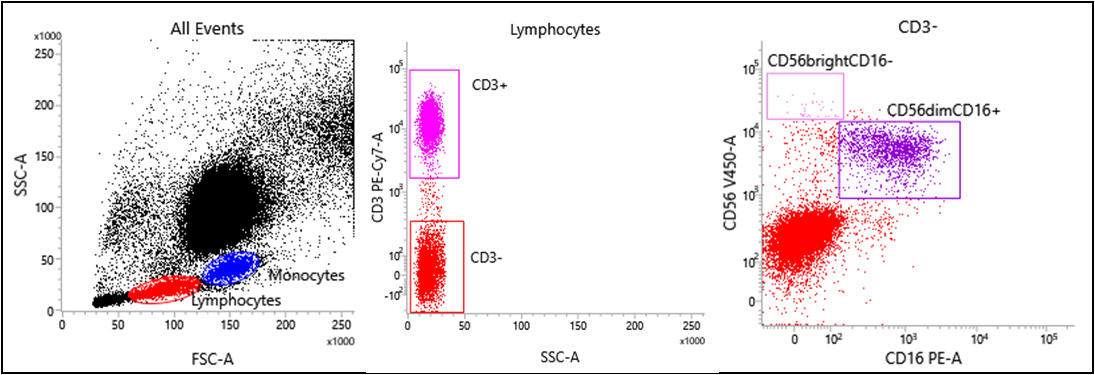


**Supplementary figure 4.** Within lymphocyte gate, NK cells were identified as CD3- lymphocytes and divided in immature NK cells (CD56^bright^CD16-, in pink) and mature NK cells (CD56^dim^CD16+, in purple).


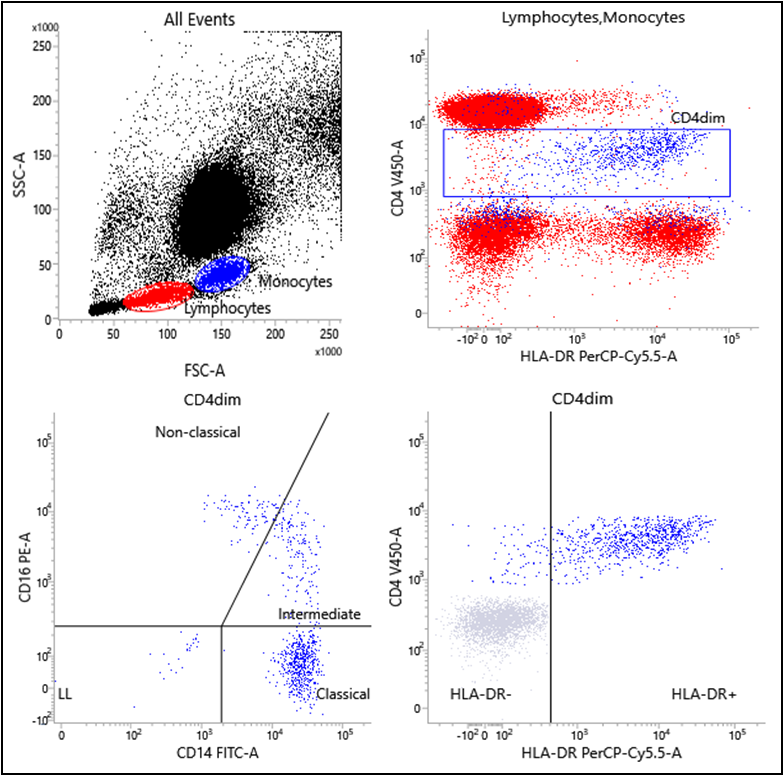


**Supplementary figure 5.** Monocyte Subpopulations. Monocytes (blue) were firstly gated on the basis of their scatter properties (Side Scatter, SSC and Forward Scatter, FSC) and subsequently refined on the basis of their CD4^dim^ expression in a CD4 vs HLA-DR dot plot, taking lymphocytes events (red) as positive/ negative internal control. Total monocyte population (CD4^dim^) was then classified according to their CD14 and CD16 expression in classical (CD14+CD16-), intermediate (CD14+CD16+) and non-classical monocytes (CD14^dim^CD16+); expression of HLA-DR was also evaluated (negative population in grey).

**Supplementary figure 6**. In a subgroup of 24 patients we evaluated the correlation between IL-6 serum values and monocytes highlighting how the two variables were not correlated. (R= linear regression; p= p value).


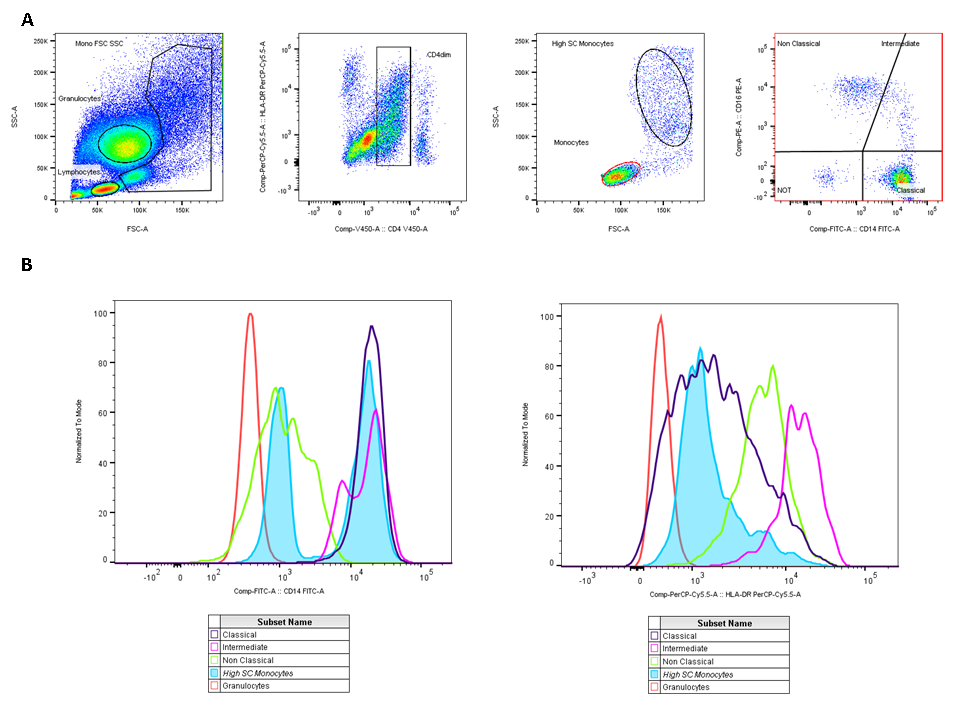


**Supplementary figure 7.** **A)** Gating strategy for granulocytes, High-SC monocytes and monocyte subpopulations analysis. **B)** Histogram overlay showing comparison of CD14 and HLA-DR MFI between granulocytes (red line), high-SC (*(*filled light blue line), non classical (green line), intermediate (violet line) and classical monocytes (pink line).

## Supplementary Table

**Supplementary table 1.** List of the employed antibodies.

|  | **Antigen** | **Clone** | **Fluorochrome** |
| --- | --- | --- | --- |
| **i) Multitest 6-color TBNK commercial Kit BD Cat.337166** | | | |
| **iii) CD4+ T-cells**  **polarization** | CD45RA | L48 | FITC |
|  | CCR7 | 150503 | PE |
|  | CD4 | SK3 | PerCP-Cy5.5 |
|  | CD25 | M-A251 | PE-Cy7 |
|  | CCR6 | 11A9 | AlexaFluor647 |
|  | CD8 | SK1 | APC-Cy7 |
|  | CD3 | UCHT1 | V450 |
|  | CD127 | HIL-7R-M21 | BV510 |
|  | CXCR3 | 1C6/CXCR3 | BV605 |
| **iii-iv) T Lymphocyte Activation and Monocytes** | CD14  CD16 | MΦP9  B73.1 | FITC  PE |
|  | HLA-DR | L243 | PerCP-Cy5.5 |
|  | CD3 | SK7 | PE-Cy7 |
|  | CD38 | HB-7 | APC |
|  | CD8 | SK1 | APC-Cy7 |
|  | CD4 | SK3 | V450 |
| **v) NK** | CD16 | B73.1 | PE |
|  | CD3 | SK7 | PE-Cy7 |
|  | CD56 | B159 | V450 |
